# Characterization of the genomic sequence of a circo-like virus and of three chaphamaparvoviruses detected in mute swan (*Cygnus olor*)

**DOI:** 10.1128/mra.01186-23

**Published:** 2024-02-20

**Authors:** Sarah François, Sarah C. Hill, Christopher M. Perrins, Oliver G. Pybus

**Affiliations:** 1 Department of Biology, University of Oxford, Oxford, United Kingdom; 2 DGIMI, Univ Montpellier, INRAE, Montpellier, France; 3 Department of Pathobiology and Population Science, Royal Veterinary College, Hatfield, United Kingdom; Katholieke Universiteit Leuven, Belgium

**Keywords:** wildlife, swan, waterbird, surveillance, viral metagenomics, virus discovery, parvovirus, CRESS DNA virus

## Abstract

We report the complete genomes of four ssDNA viruses: a circular replication-associated protein-encoding single-stranded DNA virus belonging to a clade previously detected only in mammals, and three chaphamaparvoviruses, which were detected by viromic surveillance of mute swan (*Cygnus olor*) fecal samples from the United Kingdom.

## ANNOUNCEMENT

Our knowledge of viruses infecting wild birds remains scarce, which is detrimental to poultry health and wildlife conservation (1, 2).

We processed seven mute swan (*Cygnus olor*) non-invasive samples collected in United Kingdom between 2016 and 2019 [for details, see reference (3)]. About 0.5 mL of feces was collected into a tube containing 1 mL of Universal Transport Media. Tubes were shaken and kept on ice in the field, and stored at −80°C.

Viromes were obtained as described in reference (4). We followed manufacturers’ instructions and default parameters except where otherwise noted. Samples were homogenized by a bead beater, filtered through a 0.45 µm filter, digested by DNaseI and RNaseA incubation at 37°C for 1.5 h. DNA and RNA were extracted using a QIAamp Viral RNA Mini Kit. Reverse transcription was performed using a SuperScript IV VILO kit, cDNAs were purified by a QIAquick PCR Purification Kit, and dsDNA was synthesised by Klenow DNA polymerase I. DNA was amplified by random PCR amplification (Q5 Hot Start High-Fidelity kit). PCR products were purified using a NucleoSpin gel and PCR clean-up kit. Libraries were prepared using a NEB NEXT Ultra II DNA Library prep kit, and sequenced on a NovaSeq6000 in 2 × 150 bp paired-end mode.

Adaptors were removed and reads were filtered for quality (q30 and length >45 nt) using cutadapt 2.19 (5), and 153,109,590 paired-end reads were assembled into contigs by MEGAHIT 1.2.9 (6). Taxonomic assignment was achieved using DIAMOND 0.9.30 against the NCBI nr protein database (7). Genome coverage was assessed by mapping using Bowtie2 3.5.1 (local sensitive) (8). Open reading frames (ORFs) were identified using ORF finder (length cutoff >300 nt) on Geneious Prime 2022.0.2 (9), and were annotated by blastp query-centered alignment against RefSeq viral database on 18 September 2023.

**Fig 1:**
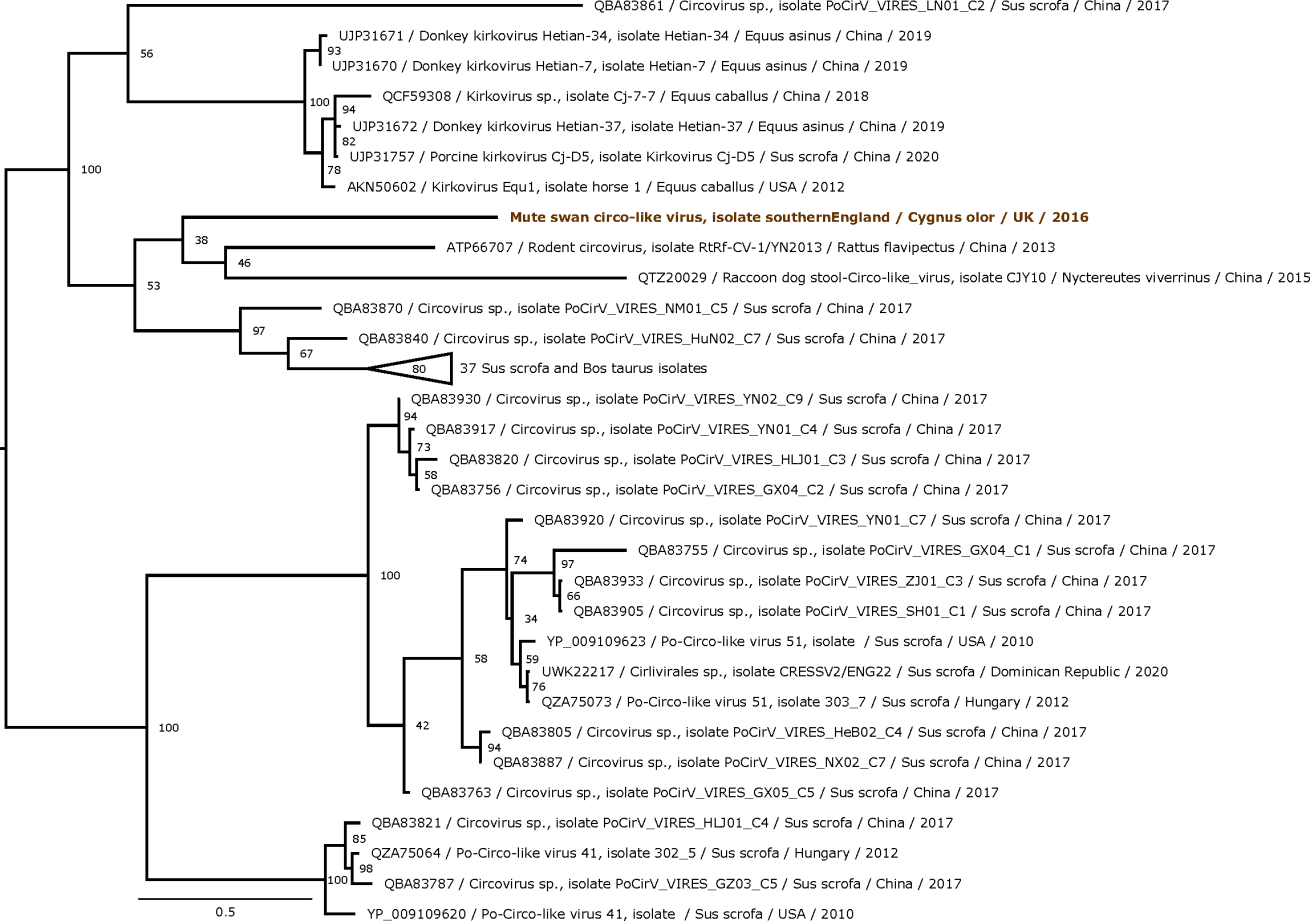
Maximum likelihood phylogenetic tree based on the capsid protein of the MSCLV and its 65 closest relatives. Protein sequences used in phylogenetic analyses were obtained by blastx from the NCBI nr database (18 September 2023). Proteins were aligned using MAFFT 7.450 with the L-INS-i algorithm. Maximum likelihood trees were estimated using RAxML 8.2.11, under the LG + G + I + F protein evolution model. Branch support was evaluated using 100 bootstrapped replicates. Trees were mid-point rooted and visualized with MEGAX 10.2.6. Bootstrap values (100 replicates) >30% are indicated at each node. The scale bar corresponds to expected amino acid substitutions per site. The sequence obtained from our sample is in bold red.

We reconstructed the complete circular genome of mute swan circo-like virus (MSCLV; length: 3,663 nt; GC content: 35.6%; average coverage depth: 298; 9,968 mapped reads, SRR26091305) and confirmed it through Sanger sequencing of PCR amplicons using GoTaq HotStar kit with overlapping primers. Chromatograms were checked for disparities. MSCLV genome contained a replication-associated protein gene (918 nt – predicted amino acid sequence: 306 aa), a capsid protein gene (507 nt – 169 aa), and a putative origin of replication marked by a conserved nonamer motif (TACTAAAGTA) flanked by a stem-loop structure (10). The closest relatives of MSCLV are pig-infecting circo-like viruses (11) [Po-Circo-like virus isolate CZH12 (MW881210) with which MSCLV shared 50.8% replication-associated protein pairwise identity; and Po-Circo-like virus HN39-01 (OP302752), 28.4% capsid protein identity] (Fig. 1). Based on the most conserved species demarcation threshold for circular replication-associated protein-encoding single-stranded DNA virus families (i.e., 77% genome-wide identity), MSCLV putatively belongs to a divergent species (12).

We report the complete CDS (coding sequence) of three members of the mammal and bird infecting *Chaphamaparvovirus* genus (*Parvoviridae* family, *Hamaparvovirinae* subfamily, 10.6084 /m9.figshare.24777786). Their closest relatives are bird-associated chaphamaparvoviruses from wild Anatidae samples, with which they shared between 50.5% and 79.6% non-structural protein 1 (NS1) protein identity (Table 1). Based on the *Parvoviridae* family species demarcation threshold (i.e., 85% NS1 protein identity), these viruses could belong to novel species (13).

**TABLE 1:** Information on the three chaphamaparvoviruses reconstructed from mute swan viromic data

Virus	Genome		Coverage			Putative proteins			Closest identified relatives			
	Size (nt)	%GC	Average	Number of reads	Sample	Name	Size (nt)	Size (AA)	Virus name	Accession number	AA pairwise identity	Host name
Chaphamaparvovirus anseriform7	4,370	41.9	50	2,019	SRR26091311	NS1	2,007	669	*Aegithalos caudatus parvoviridae* sp.	QTE03727	79.60%	*Cygnus atratus*
						NS2	594	198	Wood duck chaphamaparvovirus	QMI57945	73.20%	*Chenonetta jubata*
						NS3	438	146	Chestnut teal chaphamaparvovirus 1	YP_010802862	68.30%	*Anas castanea*
						VP	1,671	557	*Cygnus atratus* Chaphamaparvovirus	QTE04016	61.90%	*Cygnus atratus*
Chaphamaparvovirus anseriform8	4,296	39.5	230	9,206	SRR26091304	NS1	2,052	684	*Parvoviridae* sp.	QKE54873	50.50%	Unspecified bird
						NS2	621	207	Chestnut teal chaphamaparvovirus	QMI57883	50.50%	*Anas castanea*
						NS3	429	143	Chestnut teal chaphamaparvovirus	QMI57870	49.30%	*Anas castanea*
						VP	1,626	542	*Parvoviridae* sp.	QKE54874	45.90%	Unspecified bird
Chaphamaparvovirus anseriform9	4,432	39.5	206	8,343	SRR26091311	NS1	2,007	669	Mute swan feces-associated chapparvovirus 6	QUS52585	72.60%	*Cygnus olor*
						NS2	606	202	Chestnut teal chaphamaparvovirus 1	QMI57883	62.10%	*Anas castanea*
						NS3	447	149	Chestnut teal chaphamaparvovirus 1	YP_010802862	66.00%	*Anas castanea*
						VP	1,689	563	Mute swan feces-associated chapparvovirus 6	QUS52584	69.90%	*Cygnus olor*

## Data Availability

The genomic sequences of mute swan circo-like virus (MSCLV), Chaphamaparvovirus anseriform7, Chaphamaparvovirus anseriform8, and Chaphamaparvovirus anseriform9 have been deposited at GenBank under the accession numbers OR583913, OR583914, OR583915, and OR583916. High-throughput sequencing reads and raw Sanger reads were deposited in SRA under the accession no. SRR26091304 to SRR26091311 and SRR27606811 under PRJNA685791 BioProject.
